# Urate-lowering agents for asymptomatic hyperuricemia in stage 3 – 4 chronic kidney disease: Controversial role of kidney function

**DOI:** 10.1371/journal.pone.0218510

**Published:** 2019-06-17

**Authors:** Hee Jung Jeon, Jieun Oh, Dong Ho Shin

**Affiliations:** Department of Internal Medicine, Kangdong Sacred Heart Hospital, Hallym University College of Medicine, Seoul, Republic of Korea; University of Mississippi Medical Center, UNITED STATES

## Abstract

Because the serum uric acid level increases as the glomerular filtration rate (GFR) decreases, hyperuricemia is associated with chronic kidney disease (CKD). Although hyperuricemia is a risk factor for CKD progression, the causal role of uric acid remains controversial in patients with CKD and asymptomatic hyperuricemia. This study included 588 patients with stage 3–4 CKD and asymptomatic hyperuricemia. Using propensity score matching, 165 pairs treated and untreated with pharmacologic urate-lowering therapy were matched. Kaplan-Meier curves were constructed to determine the effect of urate-lowering agents on kidney survival. The prognostic value for kidney survival was ascertained using Cox regression analysis. The GFR changes over time between the patients treated and untreated with urate-lowering agents were assessed using a linear mixed model analysis. The mean age of the matched patients was 63.2 ± 12.7 years, and 52 (15.8%) patients had diabetic nephropathy. The mean estimated GFR (eGFR) and serum uric acid level were 36.7 mL/min/1.73 m^2^ and 7.8 mg/dL, respectively. During a mean follow-up period of 41.9 months, 87 developed end-stage kidney disease (ESKD). The incidence rates of ESKD were comparable between the patients treated and untreated with urate-lowering agents. The Kaplan-Meier analysis indicated that kidney survival was also comparable between them. In the multivariate analysis, heart failure and low eGFR were the significant prognostic factors for kidney survival. However, pharmacologic urate-lowering therapy was not predictive of kidney survival. The overall GFR decline rate was also comparable between the groups (*P* = 0.13). The efficacy of pharmacologic urate-lowering therapy in delaying CKD progression remains controversial. Therefore, further randomized controlled trials are needed to confirm its efficacy in attenuating kidney function deterioration in patients with stage 3–4 CKD.

## Introduction

Uric acid is the poorly soluble circulating end-product of purine metabolism in humans owing to the loss of uricase activity [1]. It has an excellent antioxidant capacity and is necessary for induction of type 2 immune responses [2]. On the contrary, the pathogenic potential of uric acid is associated with inflammatory arthritis, gout, and progression of metabolic syndrome [2]. The kidney plays an important role in uric acid excretion through a complex process involving filtration, reabsorption, and tubular secretion [3]. Generally, decreased kidney function causes hyperuricemia due to decreased excretion of uric acid. In fact, in patients with advanced-stage chronic kidney disease (CKD), the prevalence rate of hyperuricemia is known to exceed 60% [4, 5]. Recent experimental findings attracted new interest in the pathogenic role of uric acid in endothelial dysfunction, inflammation, and vascular disease [6]. In addition, several studies have reported that uric acid accelerates systemic and glomerular hypertension, leading to kidney failure by worsening glomerulosclerosis and tubulointerstitial disease [7]. Interestingly, epidemiologic studies on the general population and patients with CKD showed that hyperuricemia is a major risk factor for the development and progression of kidney diseases [8–12].

Although hyperuricemia is strongly associated with the development of CKD, whether it plays a role in the deterioration of kidney function or is only a marker reflecting such deterioration remains controversial in patients with CKD. In this context, some epidemiologic studies showed no relationship between hyperuricemia and deterioration of kidney function [5, 13]. On the contrary, several clinical trials showed the efficacy of pharmacologic urate-lowering therapy on attenuation of kidney failure in patients with CKD and hyperuricemia [14–16]. However, these studies were mostly single-center studies, which had only small numbers of patients and limited follow-up period durations. Although several meta-analyses were conducted to overcome these limitations [17, 18], the results were inconclusive because of significant heterogeneity with respect to design, end-point, and follow-up period.

The objective of this long-term observational study is to explore the effect of pharmacologic urate-lowering therapy on the kidney function of patients with CKD and asymptomatic hyperuricemia via propensity score matching.

## Materials and methods

### Ethics statement

This study was performed in accordance with the Declaration of Helsinki principles and approved by the Institutional Review Board (IRB) of Kangdong Sacred Heart Hospital (Ref. no. 2019-01-005). This was a retrospective medical record-based study, and the study subjects were de-identified. The IRB waived the need for written consent from the participants.

### Patients

**Fig 1:**
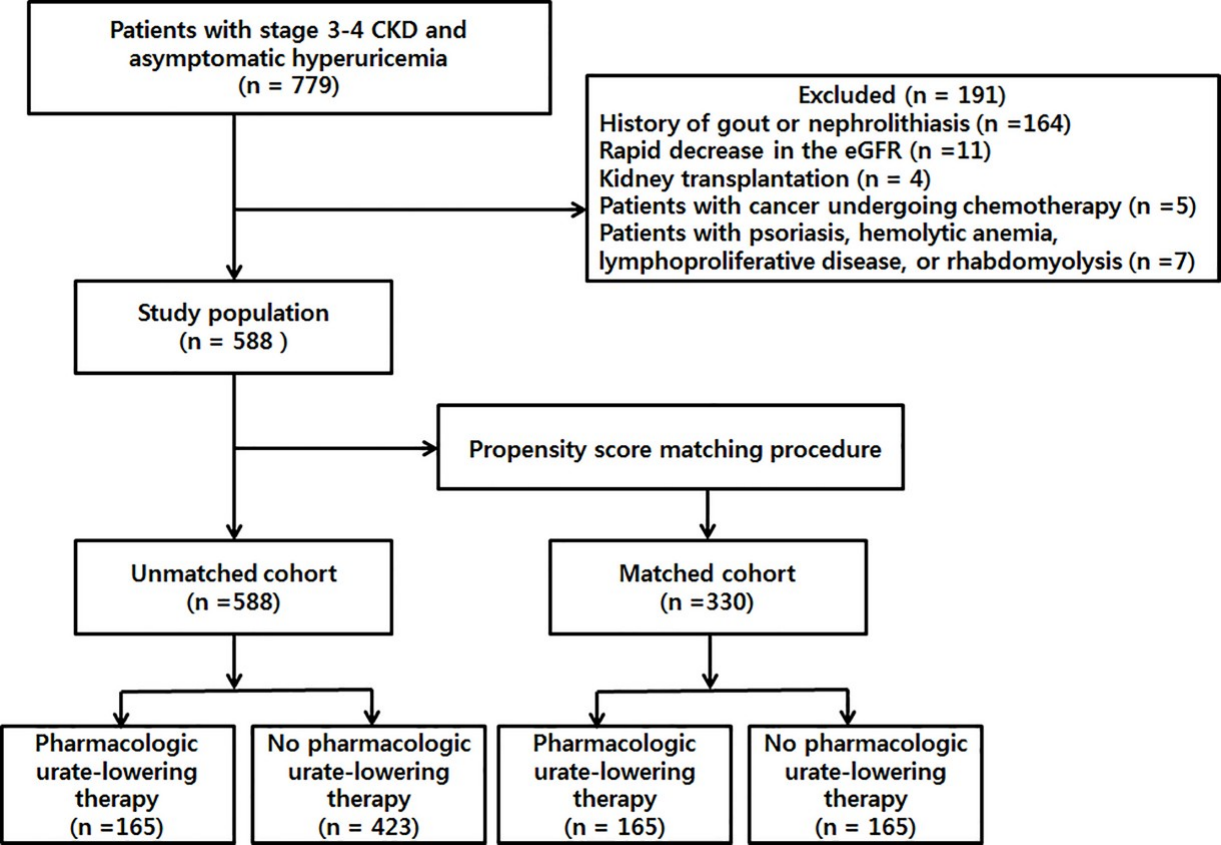
Patient selection and study flow.

The inclusion criteria were as follows: 1) age of ≥ 18 years; 2) estimated glomerular filtration rate (eGFR) of 15 to 60 mL/min/1.73 m^2^ (calculated using the four-variable Modification of Diet in Renal Disease study equation [19]) for > 3 months; 3) hyperuricemia (serum uric acid levels of ≥ 7 mg/dL); 4) naivety to pharmacologic urate-lowering therapy at least 1 year before study enrollment; and 5) follow-up period of over 12 months. Seven hundred seventy-nine patients were recruited from Kangdong Sacred Heart Hospital for the study from January 2006 to December 2018. However, subjects were excluded if there were any of the following criteria: 1) history of gout or nephrolithiasis (n = 164); 2) rapid decrease in the eGFR of at least 50% within 3 months before eligibility confirmation (n = 11); 3) previous kidney transplantation (n = 4); 4) patients with cancer undergoing chemotherapy (n = 5); and 5) conditions related to hyperuricemia not associated with decreased kidney function (e.g., psoriasis, hemolytic anemia, lymphoproliferative disease, or rhabdomyolysis) (n = 7). Thus, 588 patients were finally included in this study (Fig 1).

### Data collection

The date of the first occurrence of a serum uric acid level of > 7 mg/dL was defined as the index date. The inclusion and exclusion criteria were applied on the basis of the index date. To evaluate the effect of long-term hyperuricemia on kidney function, the serum uric acid levels were also investigated during the follow-up period. To capture longitudinal changes in serum uric acid, the area under the serum uric acid level-time curve was calculated and adjusted on the basis of the total follow-up period [20]. In other words, at each visit point in the follow-up period, visit time and serum uric acid level were multiplied, and the sum of the values was divided by the sum of the visit times. This value was defined as the actual serum uric acid level. Urate-lowering agents included xanthine oxidase inhibitors, such as allopurinol and febuxostat. Patients who received urate-lowering agents, renin-angiotensin-aldosterone system blockers, calcium channel blockers, diuretics, salicylates, and warfarin were defined as those treated for > 6 months. Proteinuria was defined as the excretion of > 0.15 of spot urine protein to creatinine ratio [21]. Heart failure was defined as either any diastolic heart failure or a systolic ejection fraction of < 40% as reported on echocardiogram [22, 23].

### Description of pharmacologic urate-lowering therapy

Among eligible patients followed-up in nephrology clinic at Kangdong Sacred Heart Hospital, the decision to treat with urate-lowering agents was made by nephrologists. Patients in treated group received urate-lowering agents from the index date. Urate-lowering agents were titrated or switched to other alternative drugs for reaching the target uric acid level (< 6 mg/dL).

### Outcome assessment

The initial study endpoint was the development of end-stage kidney disease (ESKD), defined as a condition needing maintenance dialysis or kidney transplantation. Meanwhile, the changes in the eGFR over time were evaluated according to whether the patients were treated with urate-lowering agents. In addition, subgroup analyses of eGFR decline slopes were conducted for sex, age, diabetes, CKD stage, heart failure, proteinuria, and serum uric acid level.

### Statistical analysis

Statistical analyses were performed using the SPSS 19.0 (SPSS Inc., Chicago, Illinois, USA) and R version 3.5.1 (http://cran.r-progect.org/). The Kolmogorov-Smirnov test was used to analyze the normality of parameter distribution for continuous variables. Continuous variables were expressed as means ± standard deviations or medians (interquartile ranges) and categorical variables as numbers (percentages). Propensity score matching was performed using the MatchIt package in R and variables collected at the index date. Patients with the nearest propensity scores in the two groups (patients treated and untreated with urate-lowering agents) were matched using a 1:1 scheme without replacement using a greedy algorithm. Greedy algorithm is a simple, intuitive algorithm that is used in optimization problems. The algorithm makes the optimal choice at each step as it attempts to find the overall optimal way to solve the entire problem. Propensity score matching allowed the comparison of outcomes between individuals with a similar likelihood. Differences between the two groups were assessed using the t-test, Wilcoxon’s rank sum test, and chi-squared test. Standardized mean differences were also estimated to evaluate the pre-match and post-match imbalance. Kidney survival curves were generated using the Kaplan-Meier method, and between-group survival was compared using the log-rank test. The prognostic values for kidney survival were analyzed by performing Cox regression analysis. The slope of the decline in kidney function over time was calculated using a linear regression analysis of the serial eGFR for each patient. The changes in the eGFR over time were compared between the two groups using a linear mixed model analysis. In this analysis, the patients treated and untreated with urate-lowering agents and follow-up period were treated as fixed effects, and each patient was treated as a random effect such that each subject had a unique intercept and slope. All probabilities were two-tailed, and the level of significance was set at 0.05.

## Results

### Patient characteristics

The baseline characteristics of the patients treated and untreated with urate-lowering agents are shown in Table 1. A total of 588 patients were included in the study, 165 (28.1%) of whom were treated with urate-lowering agents. The patients treated with urate-lowering agents had a higher proportion of men, higher age, longer follow-up period, and higher serum uric acid level than those untreated with such. After propensity score matching, the two groups were found to have similar characteristics. Of note, standardized mean differences before and after propensity score matching were represented in S1 Fig. Of the 330 patients in the matched analysis, 254 (77.0%) were men, and the average patient age was 58.2 ± 13.6 years. The mean eGFR and serum uric acid level were 36.5 ± 14.9 mL/min/1.73 m^2^ and 7.8 ± 8.2 mg/dL, respectively. In addition, the underlying cause of CKD was diabetes in 52 (15.8%) patients.

**Table 1:** Characteristics of the study population before and after propensity score matching.

	Before propensity score matching			After propensity score matching		
	Patients treated with pharmacologic ULT	Patients untreated with pharmacologic ULT	*P* value	Patients treated with pharmacologic ULT	Patients untreated with pharmacologic ULT	*P* value
	(n = 423)	(n = 165)		(n = 165)	(n = 165)	
Men (%)	245 (57.9)	131 (79.4)	<0.001	123 (74.5)	131 (79.4)	0.36
Age (year)	65.6 ± 13.3	57.6 ± 14.0	<0.001	58.8 ± 13.2	57.6 ± 14.0	0.43
Follow-up (month)	30.1. ± 23.3	44.0 ± 33.4	<0.001	39.8 ± 27.9	44.0 ± 33.4	0.22
Cause of CKD						
Diabetes (%)	97 (22.9)	26 (15.8)	0.06	26 (15.8)	26 (15.8)	1.00
Non-diabetes (%)	329 (77.1)	139 (84.2)		139 (84.2)	139 (84.2)	
Comorbid conditions						
Heart failure	138 (32.6)	89 (32.1)	0.99	55 (33.3)	53 (32.1)	0.91
Myocardial infarction	26 (6.1)	9 (5.5)	0.90	10 (6.1)	9 (5.5)	0.65
Proteinuria						
Absent	107 (25.3)	38 (23.0)	0.57	38 (23.0)	37 (22.4)	0.90
Present^a^	316 (74.7)	127 (77.0)		127 (77.0)	128 (77.6)	
Laboratory parameters						
eGFR (mL/min/1.73 m^2^)	36.1 ± 14.1	37.0 ± 14.6	0.51	35.9 ± 15.1	37.0 ± 14.6	0.52
Stage 3 CKD (%)	252 (59.6)	102 (61.8)	0.62	93 (56.4)	102 (61.8)	0.31
Stage 4 CKD (%)	171 (40.4)	63 (38.2)		72 (43.6)	63 (38.2)	
Urine p/cr	0.57 (0.14–1.95)	0.77 (0.17–1.84)	0.52	0.80 (0.17–2.61)	0.79 (0.18–1.84)	0.66
Serum uric acid level (mg/dL)	7.6 ± 0.6	7.9 ± 0.9	<0.001	7.7 ± 0.7	7.9 ± 0.9	0.07
Medications						
RAAS blocker (%)	282 (66.7)	130 (78.8)	0.01	131 (79.4)	130 (78.8)	0.89
CCB (%)	159 (37.6)	74 (44.8)	0.13	76 (46.1)	74 (44.8)	0.91
Diuretics (%)	99 (23.4)	55 (33.3)	0.02	58 (35.2)	55 (33.3)	0.82
Salicylates (%)	73 (17.3)	51 (30.9)	<0.001	50 (30.3)	51 (30.9)	0.91
Warfarin (%)	4 (0.9)	3 (1.8)	0.65	3 (1.8)	3 (1.8)	1.00

Notes: Values are expressed as medians ± standard deviations or numbers (percentages). Stage 3 CKD, eGFR of 60–30 mL/min/1.73 m^2^; stage 4 CKD, eGFR of 15–30 mL/min/1.73 m^2^; diuretics, thiazide or furosemide

Abbreviations: CCB, calcium channel blocker; CKD, chronic kidney disease; eGFR, estimated glomerular filtration rate; RAAS, renin-angiotensin-aldosterone system; ULT, urate-lowering therapy; Urine p/cr, spot urine protein to creatinine ratio

^a^Spot urine protein to creatinine ratio of > 0.15

### Details of pharmacologic urate-lowering therapy after propensity score matching

Ninety patients were prescribed with allopurinol as the urate-lowering agent. The starting dose of allopurinol ranged from 50 mg/day to 100 mg/day, with 100 mg/day being the most common (55/90, 61.1%). The maintenance dose of allopurinol was 100 mg in 65 patients, 200 mg in 23 patients, and 300 mg in two patients. Thirty-four patients switched to febuxostat with a maintenance dose of 40 mg/day. The reason for discontinuation of allopurinol was inefficacy or presence of side effects. Meanwhile, 75 patients were prescribed with febuxostat. The starting dose of febuxostat ranged from 20 mg/day to 40 mg/day, with 20 mg/day being the most common (49/75, 65.3%). The maintenance dose of febuxostat was 40 mg in 71 patients and 80 mg in four patients.

### Serum uric acid levels after propensity score matching

The baseline serum uric acid levels were comparable between the patients treated and untreated with urate-lowering agents. However, the final serum uric acid level (5.9 ± 1.6 vs. 8.1 ± 1.4, *P* < 0.001) and actual serum uric acid level (6.1 ± 1.0 vs. 8.0 ± 1.0, *P* < 0.001) were significantly lower in the patients treated with urate-lowering agents than in those untreated with such. Among the patients treated with urate-lowering agents, the percentage of those whose serum uric acid levels decreased to 6.0 mg/dL during the first 3 months was 98.2%.

### Incidence rate of ESKD after propensity score matching

**Fig 2:**
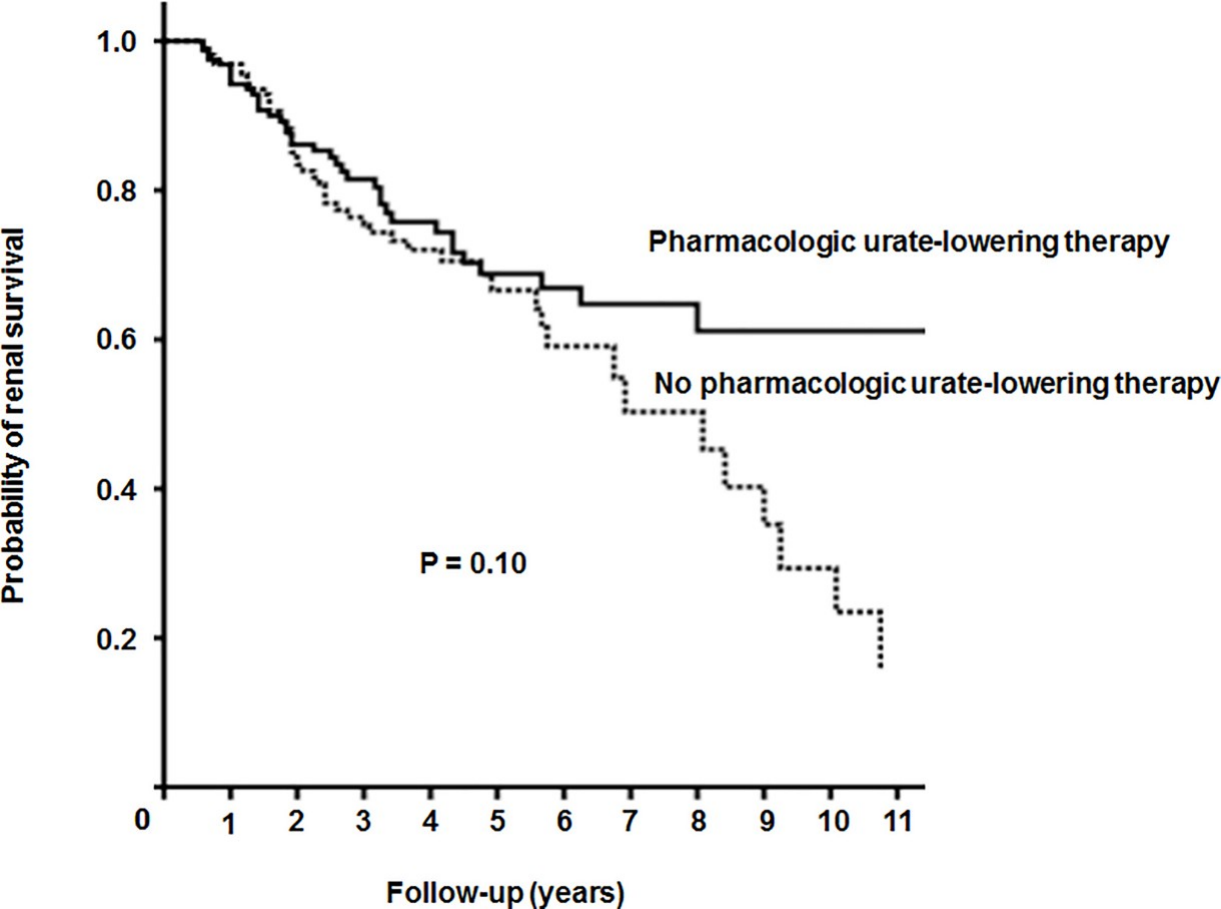
Kaplan-Meier analysis of the cumulative kidney survival of the patients with asymptomatic hyperuricemia. The dotted line and the solid line depict the patients treated and untreated with pharmacologic urate-lowering therapy, respectively.

During a mean follow-up period of 41.9 ± 30.8 months, 87 patients developed ESKD. The incidence rates of ESKD were comparable between the patients treated and untreated with urate-lowering agents (38; 23.0% vs. 49; 29.7%, *P* = 0.21). The Kaplan-Meier analysis indicated that kidney survival was also comparable between them (Fig 2). The Cox regression analysis was conducted to evaluate the prognostic values for kidney survival. In the univariate analysis, heart failure, diabetes, proteinuria, and low eGFR were the significant prognostic factors for kidney survival. In the multivariate analysis, only heart failure, proteinuria, and low eGFR were the significant prognostic factors. However, pharmacologic urate-lowering therapy was not predictive of kidney survival (Table 2).

**Table 2:** Hazard ratios for end-stage kidney disease after propensity score matching.

	Univariate		Multivariate	
Variables	Hazard ratio (95% CI)	*P* value	Hazard ratio (95% CI)	*P* value
Men (vs. women)	1.09 (0.67–1.78)	0.74		
Age (year)	0.99 (0.97–1.00)	0.07		
Diabetes (vs. non-diabetes)	2.12 (1.29–3.47)	0.003	1.51 (0.90–2.54)	0.12
Heart failure (vs. non-heart failure)	1.76 (1.15–2.70)	0.01	1.73 (1.12–2.69)	0.01
Proteinuria^a^ (vs. absent)	30.29 (4.22–217.53)	0.001	13.74 (1.89–100.06)	0.01
eGFR (mL/min)	0.93 (0.91–0.95)	<0.001	0.94 (0.92–0.96)	<0.001
Serum uric acid level (mg/dL)	1.09 (0.84–1.40)	0.53		
Urate-lowering therapy	0.70 (0.46–1.08)	0.1		
RAAS blocker	2.91 (1.34–6.31)	0.01	1.96 (0.89–4.28)	0.09
Diuretics (thiazide or furosemide)	2.25 (1.47–3.45)	<0.001	1.17 (0.75–1.82)	0.50

Abbreviations: CI, confidence interval; eGFR, estimated glomerular filtration rate; RAAS, renin-angiotensin-aldosterone system

^a^Spot urine protein to creatinine ratio of > 0.15

### Comparison of the eGFR over time

**Fig 3:**
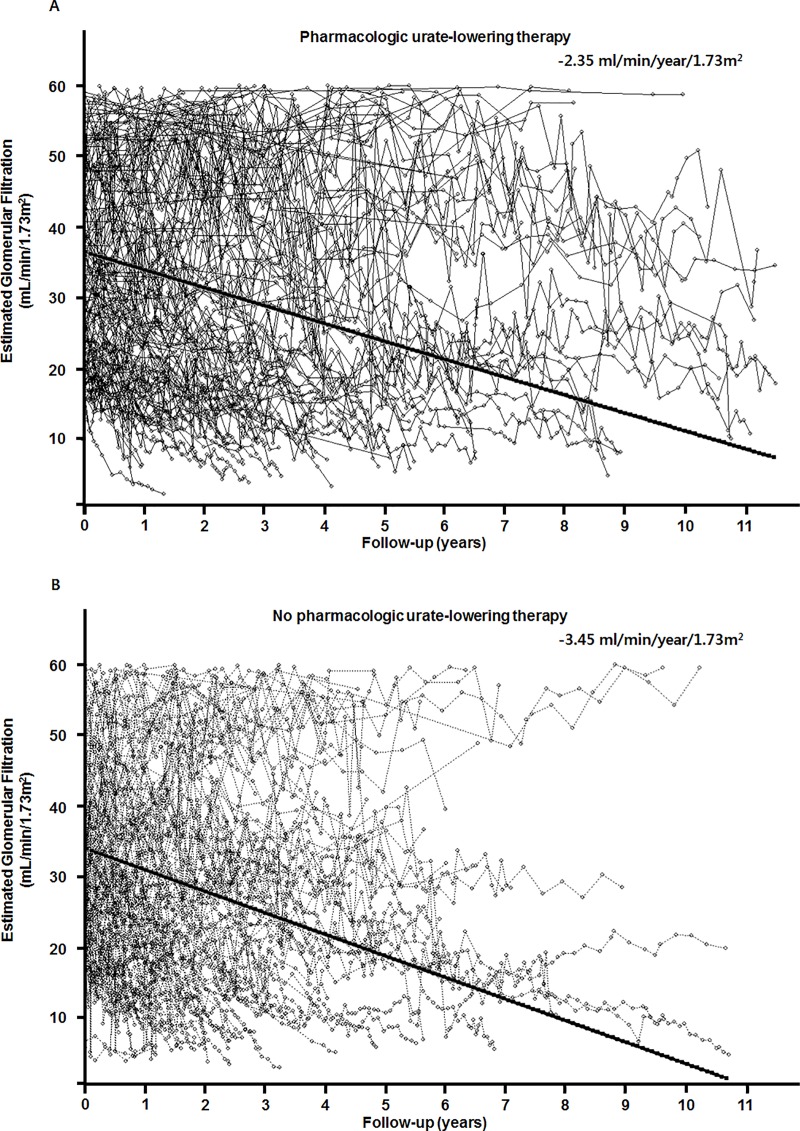
Changes in the eGFR over time in the patients with asymptomatic hyperuricemia. Patients treated with pharmacologic urate-lowering therapy (A) and those untreated with pharmacologic urate-lowering therapy (B). The dark solid lines represent the predicted values. eGFR, estimated glomerular filtration rate.

The eGFR during the follow-up period is shown in Fig 3. The linear mixed model analysis revealed that the eGFR over time was comparable between the patients treated and untreated with urate-lowering agents. The mean values of each patient’s eGFR decline slope in the two groups were also comparable (- 2.35 ± 0.39vs. - 3.45 ± 0.60 mL/min/year/1.73 m^2^, *P* = 0.13).

The subgroup analysis of the eGFR decline slope was conducted according to sex, age, heart failure, diabetes, proteinuria, CKD stage, and serum uric acid level. It revealed no significant difference between the patients treated and untreated with urate-lowering agents (Table 3).

**Table 3:** Subgroup analysis of the eGFR decline slopes.

Subgroup	Group	N	Mean	Between-group difference (95% CI)	*P* value
Sex					
Male	No pharmacologic ULT	123	- 3.15	- 0.89 (- 2.74–0.97)	0.35
	Pharmacologic ULT	131	- 2.26		
Female	No pharmacologic ULT	42	- 2.31	0.40 (- 1.71–2.52)	0.70
	Pharmacologic ULT	34	- 2.71		
Age					
< 58 years	No pharmacologic ULT	75	- 3.33	- 0.08 (- 2.82–2.66)	0.96
	Pharmacologic ULT	85	- 3.25		
≥ 58 years	No pharmacologic ULT	90	- 2.61	- 1.20 (- 2.67–0.26)	0.11
	Pharmacologic ULT	80	- 1.41		
Diabetes					
Absent	No pharmacologic ULT	139	- 2.23	- 0.01 (- 1.59–1.57)	0.99
	Pharmacologic ULT	139	- 2.22		
Present	No pharmacologic ULT	26	- 6.72	- 3.62 (- 7.61–0.37)	0.07
	Pharmacologic ULT	26	- 3.1		
Heart failure					
Absent	No pharmacologic ULT	110	- 2.93	- 0.48 (- 1.94–0.98)	0.52
	Pharmacologic ULT	112	- 2.45		
Present	No pharmacologic ULT	55	- 4.49	- 2.33 (- 5.48–0.81)	0.15
	Pharmacologic ULT	53	- 2.16		
CKD stage					
3	No pharmacologic ULT	93	- 2.50	- 0.90 (- 2.98–1.17)	0.39
	Pharmacologic ULT	102	- 1.60		
4	No pharmacologic ULT	72	- 4.7	- 1.10 (- 2.78–0.59)	0.20
	Pharmacologic ULT	63	- 3.6		
Proteinuria					
Absent	No pharmacologic ULT	38	0.15	0.06 (- 1.84–1.97)	0.95
	Pharmacologic ULT	37	0.09		
Present^a^	No pharmacologic ULT	127	- 4.53	- 1.47 (- 3.15–0.22)	0.89
	Pharmacologic ULT	128	- 3.06		
Serum uric acid level					
< 7.8 mg/dL	No pharmacologic ULT	107	- 3.74	- 1.47 (- 3.26–0.31)	0.14
	Pharmacologic ULT	114	- 2.27		
≥ 7.8 mg/dL	No pharmacologic ULT	58	- 2.90	- 0.36 (- 2.70–1.98)	0.19
	Pharmacologic ULT	51	- 2.54		

^a^Spot urine protein to creatinine ratio of > 0.15

Abbreviations: eGFR, estimated glomerular filtration rate; ULT, urate-lowering therapy

## Discussion

We evaluated the efficacy of urate-lowering agents in attenuating the deterioration of kidney function and effects on the associated clinical factors in patients with stage 3–4 CKD and asymptomatic hyperuricemia. Although clinical factors, such as diabetes, proteinuria, heart failure, and baseline eGFR, affect the deterioration of kidney function, there was no significant difference in kidney survival between the patients treated and untreated with urate-lowering agents. In addition, the subgroup analysis of the eGFR decline slope according to sex, age, heart failure, diabetes, proteinuria, CKD stage, and serum uric acid level revealed no significant difference between them.

There is accumulating evidence that hyperuricemia has a causal relationship with the deterioration of kidney function [7–12]; however, such a relationship remains debated upon. In addition, as risks of inappropriate treatment of asymptomatic hyperuricemia have been reported [24, 25], well-designed and well-conducted studies are needed to obtain definitive conclusions on the efficacy of urate-lowering agents in treating asymptomatic hyperuricemia in patients with CKD. The FEbuxostat vs placebo rAndomized controlled Trial regarding reduced renal function in patients with Hyperuricemia complicated by chRonic kidney disease stage 3 (FEATHER) study was conducted as a randomized controlled trial (RCT) to assess the effect of urate-lowering agents on the kidney function of patients with CKD and asymptomatic hyperuricemia [26]. This study was designed to investigate whether febuxostat attenuates the deterioration of the kidney function of patients with CKD and hyperuricemia without any history of gout. However, there were no significant differences in the eGFR decline slope between their febuxostat group and placebo group. In line with this finding, we clearly showed herein that the eGFR decline slopes were comparable between the patients treated and untreated with urate-lowering agents. Although the subgroup analysis in the FEATHER study demonstrated a significant benefit of preserved kidney function from febuxostat administration in patients without proteinuria, the subgroup analysis in our study revealed that there were no benefits regarding kidney function from urate-lowering agent administration. This discrepancy in the results could be explained by the following reasons. First, more patients with stage 4 CKD and proteinuria were included in our study compared with the FEATHER study. Second, different agents (i.e., febuxostat and allopurinol) were prescribed as the urate-lowering agents in our study. The Febuxostat for cerebral and caRdiorenovascular Events prEvention stuDy (FREED) was also an RCT that evaluated the effect of febuxostat in patients ≥ 65 years of age with hyperuricemia at a risk for any cerebral or cardiovascular disease [27]. This study showed that lowering the serum uric acid levels using febuxostat had a protective effect on kidney function. However, in this study, kidney failure was defined as a composite outcome, including the occurrence of proteinuria, as well as increased serum creatinine level. In addition, more patients with stage 3 CKD were also included in this study compared with our study.

It is well known that certain clinical factors, such as diabetes, proteinuria, heart failure, and baseline eGFR, could exacerbate the eGFR decline rate in patients with CKD [28–31]. In our study, although pharmacologic urate-lowering therapy was not related to kidney survival, univariate Cox regression analysis revealed that the above-mentioned clinical factors were also the significant prognostic factors for kidney survival. Especially, in multivariate Cox regression analysis, heart failure and proteinuria were found to be an independent predictor of kidney survival. Experimental findings suggested that uric acid leads to kidney failure primarily by causing systemic and glomerular hypertension [32]. In addition, recent clinical studies showed that urate-lowering therapies in asymptomatic hyperuricemia patients with CKD 2–5 have higher rates of eGFR improvement [33–35]. However, patients with advanced-stage CKD, including stages 4 and 5, commonly develop severe systemic hypertension mainly due to water and sodium retention. In this situation, the contribution of uric acid in deteriorating kidney function may become less relevant. In fact, the study by Levy G et al. showed patients on urate-lowering therapy have no improvement in the eGFR in CKD stage 4 [34]. In addition, some epidemiologic studies showed that hyperuricemia was not significantly associated with kidney survival [5, 13]. Therefore, large-scale RCTs on the treatment of hyperuricemia for kidney survival in patients with mild-to-moderate CKD, including stages 2 and 3 CKD, according to certain pathologic conditions are needed.

Xanthine oxidase inhibitors with either allopurinol or febuxostat are recommended as the first-line pharmacologic urate-lowering agents [36]. Allopurinol has been commonly used for > 50 years [37]. However, mild side effects, such as rash, leukopenia or thrombocytopenia, and diarrhea, occur in 3% - 5% of patients taking such [37]. In addition, although allopurinol hypersensitivity syndrome (AHS) is infrequent, it is a life-threatening side effect, with a mortality rate of 20% - 25%. Further, concurrent thiazide use and kidney failure increase the risk for AHS. Moreover, in the Korean population, the prevalence rate of human leukocyte antigen-B*5801 findings related to AHS is relatively high (12.2%) [36]. Meanwhile, febuxostat is well tolerated in patients experiencing hypersensitivity/intolerance to allopurinol [38]. In addition, it is metabolized predominantly in the liver, with only 1% - 6% of the dose being excreted through the kidney [39]. Therefore, the process of eliminating febuxostat from the body allows its safe administration in patients with CKD. However, the Cardiovascular Safety of Febuxostat and Allopurinol in Patients with Gout and Cardiovascular Comorbidities trial showed that the all-cause and cardiovascular mortality rates were higher in the febuxostat group than in the allopurinol group in patients with gout and cardiovascular disease [40]. Taken together, pharmacologic urate-lowering agents must be carefully administered to patients with CKD.

Our study has certain limitations. First, the study was an uncontrolled retrospective study. However, propensity score matching was used to reduce any bias owing to confounding variables commonly found in uncontrolled data. In addition, in most patients treated with urate-lowering agents, the uric acid levels were consistently maintained at < 6.0 mg/dL for a long period. Second, only a small number of patients were enrolled in this study. Therefore, the interpretation of the subgroup analysis findings may be limited. Third, different agents (i.e., febuxostat and allopurinol) were prescribed as the urate-lowering agents in our study.

In conclusion, considering the side effects of urate-lowering agents in patients with CKD, there is a need to establish their efficacy according to certain pathologic conditions in patients with stage 2–3 CKD. Therefore, a large-scale prospective trial is needed to confirm such.

## Supporting information

S1 Fig Standardized differences before and after propensity score matching comparing covariate values for patients treated with pharmacologic ULT or untreated with pharmacologic ULT. ULT, urate-lowering therapy.
(TIF)
