# Supplementary figures and images for: Urate-lowering agents for asymptomatic hyperuricemia in stage 3 – 4 chronic kidney disease: Controversial role of kidney function
Source: PLoS One. 2019 Jun 17;14(6):e0218510. doi: 10.1371/journal.pone.0218510 (PMC6576756; doi:10.1371/journal.pone.0218510)

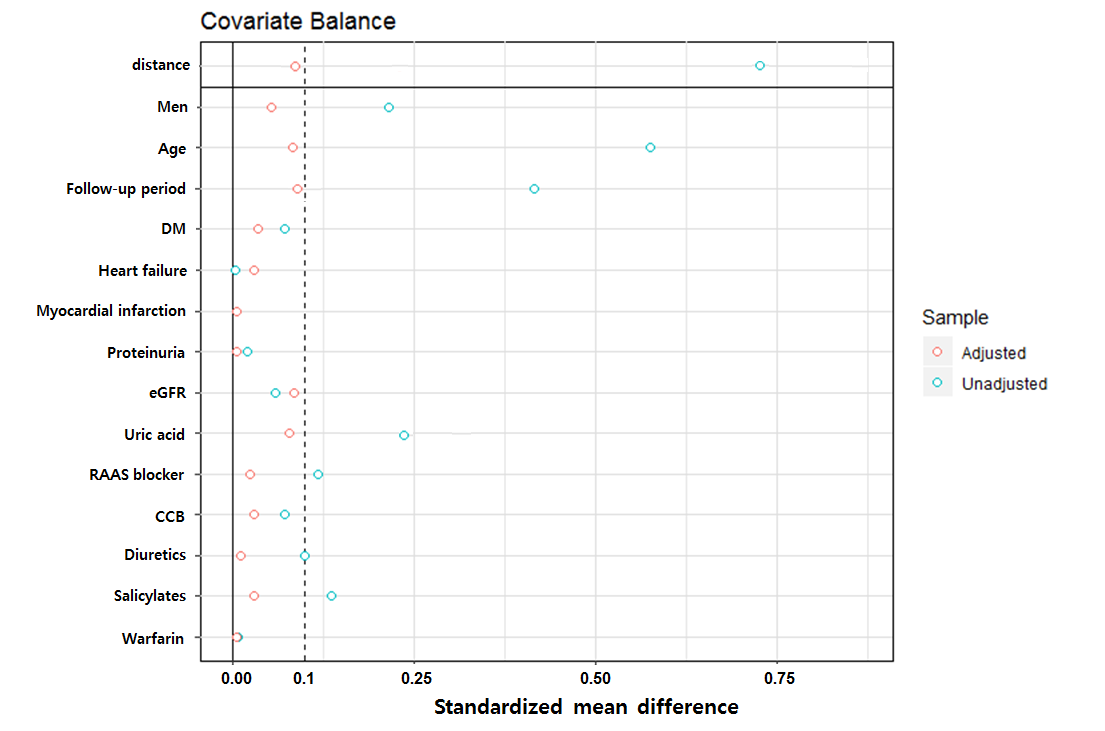

Supplement: S1 Fig — ULT, urate-lowering therapy. (TIF) [file pone.0218510.s001.tif]
